# Perceived team roles of medical students: a five year cross-sectional study

**DOI:** 10.1186/s12909-022-03263-4

**Published:** 2022-03-22

**Authors:** Anke Boone, Mathieu Roelants, Karel Hoppenbrouwers, Corinne Vandermeulen, Marc Du Bois, Lode Godderis

**Affiliations:** Centre for Environment and Health, Department of Public Health and Primary Care, University of Leuven (KU Leuven), Leuven, Belgium

**Keywords:** Teamwork, Medical Education, Curriculum, Medical students, Healthcare

## Abstract

**Introduction:**

Despite the increasing importance of teamwork in healthcare, medical education still puts great emphasis on individual achievements. The purpose of this study is to examine medical students’ team role preferences, including the association with gender and specialty; and to provide implications for policy makers and medical educators.

**Methods:**

We used an exploratory methodology, following a cross-sectional design. Data was collected from first year master students in medicine (*n* = 2293) during five consecutive years (2016–2020). The Belbin Team Role Self Perception Inventory (BTRSPI) was used to measure medical students’ self-perceptions of their team role.

**Results:**

The Team Worker was the most preferred team role among medical students (35.8%), regardless of gender or specialty. Female and male students had similar team role patterns, although female students scored higher on Team Worker (40.4% vs. 29.1%, *P* < .001) and Completer-Finisher (14.0% vs. 8.0%, *P* < .001). With regard to specialties, the Team Worker role was more often chosen by general practitioners than by person-centered and technique-oriented specialties (47.1% vs. 41.8% vs. 29.1%, *P* < .001).

**Conclusions:**

Our findings contribute to an increased scientific understanding of how medical students perceive their own team role, and how this is related to gender and specialty. This is valuable due to the increased importance of interdisciplinary teamwork in healthcare. Medical schools should prioritize stimulating teamwork skills through the implementation of different interventions at all stages (i.e. from the admission process to curricula to residency) and all levels (i.e. explicit and implicit curricula).

## Highlights

• Team worker is the most frequently preferred team role among medical students, regardless of gender or specialty.

• Female students scored higher on the Team Worker role compared to male students.

• Team Workers were more present in future general practitioners and lower in technique-oriented specialties, with students who chose a person-centered specialty in the middle.

• Medical education should stimulate teamwork skills throughout the entire educational career, from the admission process to curricula to residency .

## Introduction

The importance of teamwork in healthcare is becoming increasingly apparent [1]. Teamwork is an often used term and may be defined as the process of interactions between team members, who combine their collective resources to accomplish common goals [2, 3]. In healthcare, it is crucial that, for example, emergency teams, surgical teams or rehabilitation teams, work across these professional, disciplinary and sectoral boundaries [3].

In addition, the growing complexity of patient care and the surge in comorbidities have resulted in an increase in medical specializations (i.e. among medical doctors, nurses, psychologists, etc.) [1, 4]. To overcome specialty fragmentation, multidisciplinary healthcare teams have become part of the solution, characterized by strong collaborations between different medical specialties (e.g. general practitioners, surgeons) and health professions (e.g. medical doctors, nurses) [1].

Effective teamwork in healthcare has been found to be beneficial for a variety of additional reasons, such as increased work-engagement [5], lower burnout risk, higher performance and fewer medical errors [6]. However, it is important to note that effective teamwork becomes more challenging with the rise in specializations, as different medical specializations have distinct interests, technical jargon and educational backgrounds [4].

Evidence on medical doctors’ teamwork skills shows they are usually not considered Team Workers, but rather solo performers and independent decision-makers [7–9]. They have a tradition of working independently and are trained to take full responsibility as medical experts [7–10]. Nevertheless, former studies indicate differences in study year [11–15], gender [16, 17] and medical specialties [8]. For instance, women are usually better Team Workers compared to men, while men generally score higher on leadership [16, 17]. Additionally, certain medical specialties have a longer tradition of teamwork [8] or require higher levels of empathy [12], which is an important characteristic of teamwork [18]. For example, medical students who prefer general practice or person-centered specialties (e.g. psychiatry) usually have higher empathy scores than those who prefer technique-oriented specialties (e.g. clinical biologist) [12]. Furthermore, first year bachelor medical students seem to score higher on empathy compared to their third and fourth year counterparts, implying a potential reduction in empathy throughout medical education [12–15].

To ensure high-quality healthcare, teamwork has been increasingly identified and incorporated into curricula for medical education [19]. The Canadian Medical Educational Directives for Specialists (CanMEDS) developed an outcomes-based framework for medical education that identifies seven core competencies for medical doctors, including the Collaborator [20]. The Collaborator is described as someone who works ‘effectively with other health care professionals to provide safe, high-quality, patient-centred care’ (16, p18). Belgium, Denmark, Australia and New Zealand are among the countries that have adopted this framework as a guideline for medical education curricula [21, 22].

Despite this progress, medical education is still marked by strong competitiveness (e.g. numerus clausus) and a focus on individual achievements and outcompeting peers [10, 11, 23–25]. Evidence has indicated that medical students perceive healthcare as an individual responsibility rather than a collective one [26]. In addition, Walkiewicz et al. showed that medical students scored higher on action-oriented team roles (i.e. concerned with immediate tasks) and thinking-oriented roles (i.e. creative or analytical thinkers) than on the Team Worker role [17], which CanMEDS refers to as the Collaborator [27]. Furthermore, multiple studies have found that empathy scores decreased throughout medical education [12–15]. These findings contradict with the increasing importance of interprofessional collaboration and multidisciplinary teamwork in modern healthcare [10, 25].

There is also still much uncertainty on medical students’ perceptions with regard to their own professional team roles. We have been able to identify only one study by Walkiewicz et al. [17], but the generalizability of their findings was problematic due to the relatively small sample size. Nonetheless, examining perceptions has great value as studies have shown that people who have positive perceptions about teamwork tend to be more committed to it [28, 29]. Furthermore, perceptions and beliefs often result from past experiences, which means that providing positive experiences (i.e. on teamwork) may improve people’s commitment towards teamwork in the future [28].

The aim of this study is to examine self-perceptions of medical students on their professional team role, with a focus on teamwork. In addition, we have assessed differences according to gender and future specialty (i.e. general practice, person-centered specialty or technique-oriented specialty). Based on the literature, we expected overall higher scores for action-oriented and thinking-oriented roles versus Team Worker roles [17]; and higher scores for Team Worker roles in female students [16, 17] and in those who will later opt for a training as general practitioner or a person-oriented specialty [12].

## Materials and methods

### Participants

First year master students in medicine of the University of Leuven (KU Leuven), Belgium, were asked to complete an online questionnaire prior to starting a group assignment during five consecutive years from 2016 to 2020. The number of students enrolled per year was 487 (2016), 502 (2017), 464 (2018), 469 (2019) and 514 (2020). The total number was 2.436 students, of which 60% were female. In December of each year, designated faculty members sent the questionnaire to the students via email with a link to the survey, and a reminder was sent in January. The submission of the questionnaire was part of a curriculum activity within medical education. For the retrospective analysis of the preferred team roles’ associations with gender and specialty, ethical approval was obtained from the Social and Societal Ethics Committee of the KU Leuven (G-2020-1632). To ensure anonymity and privacy, data were pseudonymized (i.e. personal identification data were replace by a code prior to the analysis).

### Instrument

The Dutch version of the Belbin Team Role Self Perception Inventory (BTRSPI) was used to measure self-perceptions of team roles [30]. This questionnaire is based on Belbin’s Team Role model, which assesses behaviour instead of job ranking, position or personality [31]. Table 1 describes the eight different team roles according to Belbin: Completer-Finisher, Shaper, Implementer, Monitor-Evaluator, Plant, Resource-Investigator, Team Worker and Coordinator. Later, a ninth team role - the Specialist - was added based on specialist expertise. However, the Dutch BTRSPI did not include this role, and because our research primarily focused on teamwork, we included only eight Belbin team roles [31].

**Table 1:** Belbin team roles, strengths and potential weaknesses

Team role	Strengths	Potential weaknesses
**Action – Oriented Roles**		
Completer-Finisher	- Pays attention to details - Conscientiously delivers on time - Searches out lacunas	- Resistant to delegate - Worries excessively - Could exaggerate with perfectionism
Shaper	- Thrives on high pressure - Dynamic - Sets objectives	- Tendency to provocation - Could offend people - Can seem aggressive
Implementer	- Efficient and trustworthy - Hands-on mentality - Systematic and efficient	- Susceptible to inflexibility - Can be slow to embrace opportunities and changes
**Thinking – Oriented Roles**		
Monitor-Evaluator	- Sophisticated and strategic - Judges accurately - Works facts-based	- Slow decision-making - Falls short on the ability to inspire - Could be highly critical
Plant	- Highly creative and imaginative - Good problem-solving - Advances new ideas	- Might ignore expenses - Lacks effective communication - Could be easily distracted
**People – Oriented Roles**		
Resource-Investigator	- Outgoing, enthusiastic - Explores - Inquisitive nature	- Over-optimistic - Loses interest quickly - Could forget to follow up
Team Worker	- Collaborative and diplomatic - Conflict aversive - Focusses on team spirit	- Avoids confrontations - Indecisive in crisis - Hesitant to make unpopular decisions
Coordinator	- Focuses on the team - Delegates work - Mature and confident	- Might over-delegate work - Could be considered manipulative

Descriptions adapted from Belbin M., Team Roles at Work.; 2012. [31]

The questionnaire was distributed with the online survey program LimeSurvey version 2.06+ (LimeSurvey GmbH, Hamburg, Germany) for the first four years (2016–2019), and with Qualtrics (Qualtrics, Provo, UT, USA) in 2020. The BTRSPI is divided into seven categories. For each category, students have to assign a total of 10 points among eight items, based on how closely each item represents the respondent’s self-perceived behaviour. Each item relates to one of Belbin’s team roles in particular. An example is *‘I believe I can make positive contributions to a team because… [item 1/8: I am quick to see and take advantage of new opportunities].* This item corresponds with the Monitor-Evaluator. The more points a student assigns to a particular item, the better this behaviour matches the self-perceived team role. The scores which correspond to a given team role in each of the seven categories are combined, and the highest score represents the preferred team role, while the lowest score reflects the least corresponding team role [31, 32].

### Data analysis

Data were analyzed in R version 4.0 [33]. The primary outcome variable of our analysis was the preferred team role, defined as the team role with the maximum score. When multiple (usually two) team roles received the same maximum score (i.e. ties), each one of these roles was considered as ‘preferred’ such that the total prevalence is somewhat larger than 100% (i.e. 113.6%). Preferred team roles were compared separately by gender, academic year and specialty with chi-squared tests. In addition, total scores for each of the roles were compared using Spearman correlation analysis. Statistical tests with a *p*-value lower than 0.05 were considered as statistically significant.

Specialty analysis was limited to the cohorts of 2016 and 2017 since later cohorts had not yet started further training and education. For this analysis, medical specialties were grouped as General Practitioners (GP), Person-oriented Specialties (PS: internal medicine, gynecology, physical medicine and rehabilitation, pediatrics and psychiatry), and Technique-oriented Specialties (TS: surgery, neuro-surgery, orthopedic surgery, esthetical surgery, otolaryngology, ophthalmology, anesthesiology, dermatology, emergency medicine, pathology, radiography, insurance medicine, clinical biology, clinical genetics, nuclear medicine, urology and stomatology) [34].

## Results

We received 2293 answers to our online survey (global response rate 94%). The number of participants/total number of students enrolled for the course in the five cohorts was 467/487 (96%) in 2016, 475/502 (95%) in 2017, 429/464 (92%) in 2018, 457/469 (97%) in 2019 and 465/514 (90%) in 2020. The mean age of the students was 21.7 years (SD 2.0) and 60% (*n* = 1375) were female.

*Team roles* (see Table 2). The Team Worker was by far the most popular team role, receiving the highest score from 822 respondents (35.8%). The Implementer ranked second with 555 respondents (24.2%) and the Shaper third with 447 respondents (19.5%). The two least prevalent team roles were the Resource-Investigator (*n* = 54, 2.4%) and the Plant (*n* = 67, 2.9%). Differences between the five cohorts were not statistically significant (*P* > .05) (see Table 2). A total of 277 respondents (12.1%) had two or more preferred team roles, which means that they received the same highest score for more than one team role.

**Table 2:** Team roles according to the five consecutive years (2016, 2017, 2018, 2019, 2020)

	2016		2017		2018		2019		2020		Total		*P*-value
	**N**	**%**	**N**	**%**	**N**	**%**	**N**	**%**	**N**	**%**	**N**	**%**	
Completer-Finisher	45	9.6	55	11.6	44	10.3	65	14.2	56	12.0	265	11.6	0.224
Shaper	87	18.6	82	17.3	74	17.2	110	24.1	94	20.2	447	19.5	0.053
Implementer	104	22.3	126	26.5	111	25.9	110	24.1	104	22.4	555	24.2	0.425
Resource-Investigator	11	2.4	17	3.6	12	2.8	7	1.5	7	1.5	54	2.4	0.180
Team Worker	184	39.4	178	37.5	156	36.4	145	31.7	159	34.2	822	35.8	0.131
Coordinator	42	9.0	38	8.0	36	8.4	37	8.1	32	6.9	185	8.1	0.829
Monitor-Evaluator	41	8.8	47	9.9	36	8.4	39	8.5	45	9.7	208	9.1	0.903
Plant	13	2.8	12	2.5	14	3.3	13	2.8	15	3.2	67	2.9	0.960
Team roles	527	112.9	555	116.9	483	112.7	526	115	512	110.1	2603	113.6	

* *P* < .05

** *P* < .001

*Gender* (see Table 3). Female and male students showed overall similar team role patterns, while more female than male students preferred the Team Worker (40.4% vs. 29.1%, *P* < .001) and Completer-Finisher role (14.0% vs. 8.0%, *P* < .001). Male students, on the other hand, more often preferred the Monitor-Evaluator role than female students (14.1% vs. 5.7%, *P* < .001).

*Specialty (see* Table 3*).* The Team Worker was the most preferred team role among students who later opted for a training as a General Practitioner (GP) (47.1%), a Person-centered Specialty (PS) (41.8%) and a Technique-centered Specialty (TS) (29.1%). However, the frequency was largely different between disciplines, with the Team Worker role more often chosen by GPs than by PS and TS; and more often by PS than by TS (*P* < .001). Among students who opted for TS, the Team Worker role was only marginally more preferred than the Shaper or Implementer role, while Team Worker had a greater advantage on the next team role among GP and PS. Further, Shaper and Completer-Finisher were significantly more preferred team roles among TS, than among GP or PS (*P* < .05).

**Table 3:** Preferred team roles according to gender and specialty group^a^

Team roles	Female		Male		*P*-value	GP		PS		TS		*P*-value
	N	%	N	%		N	%	N	%	N	%	
Completer-Finisher	192	14.0	73	8.0	.000**	26	9.0	20	10.2	34	16.5	.028*
Shaper	253	18.4	194	21.1	.118	42	14.5	35	17.9	54	26.2	.004*
Implementer	337	24.5	218	23.7	.713	61	21.1	43	21.9	56	27.2	.256
Resource-Investigator	24	1.7	30	3.3	.027*	10	3.5	7	3.6	2	1.0	.176
Team Worker	555	40.4	267	29.1	.000**	136	47.1	82	41.8	60	29.1	3e-04**
Coordinator	100	7.3	85	9.3	.102	28	9.7	18	9.2	12	5.8	.279
Monitor-Evaluator	79	5.7	129	14.1	.000**	16	5.5	19	9.7	22	10.7	.084
Plant	28	2.0	39	4.2	.003*	8	2.8	5	2.6	2	1.0	.365

* *P* < .05

** *P* < .001

^a^*GP* general practitioner, *PS* Person-oriented Specialty, *TS* Technique-oriented Specialty. The analysis by specialty group was limited to students from 2016 and 2017

*Correlations *(see Table 4). A Spearman correlation analysis of scores given to the various team roles was conducted. This analysis indicated a moderate significant (negative) correlation between Team Worker and Shaper (r_s_ = − 0.582, *P* < .001), which means that the higher a respondent’s Team Worker score is, the lower their Shaper score. Other significant (negative) correlations appeared, although they were rather weak (r_s_ < − 0.400) [35] (e.g. Resource-Investigator vs. Completer-Finisher, Resource-Investigator vs. Implementer, Coordinator vs. Completer-Finisher, Plant vs. Shaper).

**Table 4:** Spearman correlations between team roles (above diagonal) and corresponding *p*-values (below diagonal)

	Completer-Finisher	Shaper	Implementer	Resource-Investigator	Team Worker	Coordinator	Monitor - Evaluator	Plant
Completer-Finisher		− 0.043	0.066	− 0.372	− 0.143	− 0.335	− 0.209	− 0.117
Shaper	0.041*		− 0.218	0.001	− 0.582	0.155	− 0.063	− 0.330
Implementer	0.002*	0.000**		-0.320	0.023	− 0.235	− 0.025	− 0.175
Resource-Investigator	0.000**	0.966	0.000**		− 0.079	0.119	− 0.082	0.179
Team Worker	0.000**	0.000**	0.271	0.000**		− 0.091	− 0.230	0.026
Coordinator	0.000**	0.000**	0.000**	0.000**	0.000**		− 0.100	− 0.219
Monitor- Evaluator	0.000**	0.003*	0.221	0.000**	0.000**	0.000**		− 0.064
Plant	0.000**	0.000**	0.000**	0.000**	0.220	0.000**	0.002*	

* *P* < .05

** *P* < .001

## Discussion

This explorative study shows that Team Worker is the most preferred team role among medical students, regardless of gender or specialty. Female and male students had similar team role patterns, although the former scored higher on Team Worker and Completer-Finisher. With regard to specialties, the Team Worker role was more often chosen by general practitioners than by person-centered and technique-oriented specialties; and more often by person-centered specialties than by technique-oriented specialties.

### Medical students as Team Workers

The finding that Team Worker was the most frequently preferred team role among our target group is somewhat surprising given that most studies define medical students and practicing medical doctors as independent decision-makers or solo performers [7–9, 26, 36, 37]. Therefore, we expected that medical students would score higher on action-oriented or thinking-oriented roles, than on the Team Worker role. For example, Walkiewicz et al. found that medical students scored significantly higher for action-oriented and thinking-oriented roles than for the Team Worker role, while using the same instrument (i.e. BTRSPI) [17].

The discrepancy between our findings and those of Walkiewicz et al. [17] is possibly due to their significantly smaller sample size (140 medical students) compared to 2293 medical students in our study. Other factors, such as geographical region (i.e. Poland versus Belgium), differences in medical curriculum (e.g. distinct approaches to stimulate teamwork skills), targeted grade (i.e. first and fifth grade versus fourth grade/first master; both in a 6-year bachelor-master curriculum) and study design (i.e. cross-sectional versus repeated cross-sectional) may have also contributed to the differences in results.

Further, our study examined the self-perception of team behaviour, while other studies focused on attitudes towards teamwork [36], ideas on interprofessional collaborations [37] or views on interdisciplinary team trainings [8]. These studies therefore addressed other dimensions of teamwork, as teamwork is a multi-faceted mechanism that relies on a variety of factors [38]. Consequently, our findings may complement rather than contradict former research. Medical students can prefer the Team Worker role, while at the same time being less inclined to interprofessional collaboration and perceiving themselves as independent decision-makers.

Another distinction is that our research took place relatively early in the careers of medical doctors (i.e. first year master students), as opposed to other studies that examined medical residents [36] or practicing medical doctors [7, 8]. This could mean that many medical students start their education as Team Workers, although medical education does not nurture and stimulate these qualities sufficiently. Despite the fact that our data are insufficient to corroborate this hypothesis and, hence, longitudinal studies are needed, Coulehan and Williams provide some preliminary evidence [11]. They found that parts of medical education lead to increased detachment and self-interest, even among students who started with qualities such as altruism and compassion [11]. Other studies add that first year bachelor medical students score higher on empathy compared to their third and fourth year counterparts, implying a potential reduction in empathy throughout medical education [12–15].

Whitehead found that there still exist multiple barriers to stimulating teamwork in medical education, both in the explicit (i.e. overtly introducing certain values in courses) and the implicit (i.e. hidden or informal culture) curriculum [9]. For instance, when medical educators and policy makers design and implement teamwork initiatives, they should also address hierarchical relations and medical doctors’ expert status and decision-making responsibilities. It is crucial to explore how effective teamwork can occur within these hierarchical settings, as well as how medical students can be trained to accept and share responsibilities. In addition, teamwork values trained in the explicit curriculum should be reflected in the clinical setting (implicit curriculum), because a divergence between training and clinical setting is expected to discourage teamwork [9].

### Team roles and gender

Our finding that female students score higher on the Team Worker role confirms existing literature on this topic [16, 39, 40]. Anderson and Sleap, who used the same instrument (i.e. BTRSPI), found that the Team Worker role was preferred by twice as many women as men [16]. Similarly, Wilhelmsson et al. found that female medical students displayed more positive beliefs toward teamwork than their male counterparts [39]. According to Kuhn and Villeval a possible explanation is that women are more attracted to teamwork, have more trust in their colleagues’ abilities and less confidence in their own competence, making teamwork more beneficial [40].

Furthermore, Etherington et al. found that gender may impact teamwork by undermining team morale, communication and psychological safety [41]. This might imply that interventions aimed at improving teamwork are unlikely to be effective as long as gender roles and gendered power relations remain unaddressed [41]. There is no simple solution, but medical education could start by introducing different interventions addressing gender inequity. These include, among others, genuinely recognising the problem, collecting and reporting gender data, fostering reflection on power systems, addressing implicit gender biases, introducing non-gendered parental leave, organising child care support and committing to gender equity in all policies [43]. In addition, other social factors, such as age, ethnicity, sexual orientation, religion or trainee status are equally important to be considered [41, 43].

### Team roles and future specialties

Team role preferences were not consistent across medical specialties, since the representation of Team Workers was larger in future general practitioners and lower in technique-oriented specialties, with students who chose a person-centered specialty in the middle. This gradient was expected based on former studies focusing on empathy, in which medical students choosing general practice or a person-centered specialty scored higher on empathy than people choosing technique-oriented specialties [12, 44]. It is possible that students who are more empathic are drawn to general practice or people-oriented specialties prior to their medical education. Nevertheless, Chen et al. argue that students’ career preferences may not be definitive from the start and that fostering empathy skills during medical education may have an impact on future career choices [12]. With fewer medical students opting for a career in general practice or person-centered specialties in certain regions, a greater emphasis on empathy may stimulate to meet certain societal needs [12, 45].

### Practical implications

Our findings contribute to an increased scientific understanding of how medical students perceive themselves in relation to their professional team roles, and how this is related to gender and specialty. There are several potential implications for policy makers, curriculum designers and medical teachers. First, former studies have shown that perceptions may result from experiences in the past and that people who have positive perceptions about teamwork tend to be more committed to it [28]. Hence, providing positive teamwork experiences throughout the medical curriculum may not only improve perceptions about teamwork, but also raise existing levels of commitment to teamwork.

Second, if the current medical curriculum does not sufficiently encourage teamwork and empathy skills [9, 11–13, 15, 44], the stimulation of these skills on all stages (i.e. from the admission process to curricula to residency) and all levels (i.e. explicit and implicit curricula) should be prioritized. For example, from the start medical students should be engaged in interprofessional education, where they are involved in problem-solving exercises with students from other healthcare professions (e.g. nursing or public health) [46]. With regard to the implicit curriculum, policy makers, curriculum designers and medical educators need to be aware of the existing hierarchical relations and develop strategies to empower medical students [47]. Interventions to improve teamwork are unlikely to be optimally effective when they do not take into account hierarchical relations, decision-making responsibilities or important social identity factors, such as gender or ethnicity [41]. Role models and mentors play an important part in this process through demonstration and how to behave effectively in health care teams [47]. Moreover, the provision of adequate feedback is recommended, not only on clinical knowledge, but also on teamwork and empathy skills. In addition, medical students need to be encouraged to be self-reflective on their professional team role and discuss potential role conflicts with mentors or peers [47].

Third, the development of trainings and interventions to improve teamwork throughout medical education should start from former research to ensure evidence-based interventions. For example, a recent study by Orsini et al. focused on the evaluation of two online interprofessional faculty development programs [48]. They reported three main factors that facilitate an interprofessional environment: a professions-inclusive teaching style, a flexible learning climate, and interprofessional peer work [48]. In addition, other studies on the impact of interprofessional simulated learning [42], teamwork skills modules [49], group based learning [50], problem-based learning [49] and team-based primary care [51] may include insights that are transferable to other programs seeking to enhance and support teamwork.

### Strengths and limitations

An important strength of this research includes our large sample size (*n* = 2293). We were able to collect data consistently from a large group of medical students over a five-year period, which increases the generalizability of the results. Furthermore, our research provides valuable information on medical students’ self-perceived team roles, which can be used to create new research questions or form hypotheses on cause-and-effect relationships (e.g. between medical education curricula and team work behaviour/skills). Nevertheless, we should also note several limitations of our research.

First, our study had a cross-sectional design, which might be prone to cohort effects. Nonetheless, our results have remained consistent over five consecutive years, indicating limited cohort bias. Second, the specialty analysis was limited to the cohorts of 2016 and 2017, which reduced the sample size for this research question. Third, the BTRSPI is an online questionnaire that measures students’ perceptions of their own behaviour. Self-rapportage is known to be prone to social desirability bias, although Cheung and Chan state that the ipsative scoring of the BTRSPI can reduce it [52]. Fourth, the BTRSPI’s ipsative scoring form has the disadvantage that respondents are not familiar with this type of measurement, which can result in potential errors [52]. Fifth, the questionnaire was presented to the participants as part of a group assignment during their medical education. Hence, further research is necessary to determine whether these findings can be applied and generalized to multidisciplinary healthcare teams.

## Conclusions

Given the growing importance of teamwork in our healthcare system, the results of this study are encouraging as they indicate that Team Worker is the most preferred team role among medical students. Nonetheless, a medical education system that prioritizes individual achievements over teamwork makes it difficult to adequately prepare future medical doctors for teamwork. This study suggests that increasing teamwork skills will require interventions at all stages (i.e. from the admission process to curricula to residency) and all levels (i.e. explicit and implicit curricula). If medical education succeeds in this endeavor, our future healthcare system is likely to be more collaborative, more efficient and more effective; which may result in patients receiving higher-quality care.

## Data Availability

The datasets used and/or analysed during the current study are available from the corresponding author on reasonable request.

## Abbreviations

BTRSPI: Belbin Team Role Self Perception Inventory
CanMEDS: Canadian Medical Educational Directives for Specialists
KU Leuven: University of Leuven
GP: General Practitioners
PS: Person-oriented Specialties
TS: Technique-oriented Specialties
